# 超交联多孔有机聚合物在柱固相萃取中的应用进展

**DOI:** 10.3724/SP.J.1123.2022.12003

**Published:** 2023-07-08

**Authors:** Tongtong QIN, Li GAO, Wenjie ZHAO

**Affiliations:** 河南工业大学化学化工学院,河南郑州450001; School of Chemistry and Chemical Engineering, Henan University of Technology, Zhengzhou 450001, China

**Keywords:** 超交联多孔有机聚合物, 柱固相萃取, 吸附剂, 吸附机理, hypercrosslinked porous organic polymers (HCPs), cartridge-based solid phase extraction, adsorbents, adsorption mechanism

## Abstract

超交联多孔有机聚合物(hypercrosslinked porous organic polymers, HCPs)是一类通过傅-克烷基化反应将芳香结构单元连接而制备得到的新型多孔材料,具有单体来源广泛、比表面积高、成本低廉、合成条件温和及易功能化等优点,广泛应用于气体储存、多相催化、色谱分离和有机污染物去除等领域。近年来,HCPs作为柱固相萃取吸附剂的研究较多,展现出巨大的应用潜力。基于高比表面积、优异的吸附性能、多样化的化学结构和易于化学改性等优点,HCPs材料被成功应用于不同样品基质中多种类型分析物的萃取并表现出优异的萃取性能。根据HCPs的骨架化学结构、目标分析物的性质及两者间的作用机理,我们将HCPs分为疏水型、亲水型、离子型3类,介绍了各种HCPs的特点、合成方法和在柱固相萃取中的应用。基于HCPs与分析物之间的疏水、*π-π*、亲水、氢键、离子交换等多种相互作用机理,HCPs萃取材料能够高效萃取和选择性富集不同种类的目标分析物,如苯脲类除草剂、氯酚类化合物、硝基咪唑、四环素、酸碱性药物等。将新型HCPs萃取与色谱、质谱等现代分析技术结合的方法已广泛应用于环境监测、食品安全和生化分析等领域。本文对HCPs在填充柱固相萃取中的应用进行了全面综述,并对其未来发展做出了展望。

## Abstract (en)

Hypercrosslinked porous organic polymers (HCPs), a novel type of porous materials synthesized via the Friedel-Crafts reaction, are widely used in gas storage, heterogeneous catalysis, chromatographic separation, and organic pollutant capture. HCPs have the advantages of a wide monomer source, low cost, mild synthesis conditions, and easy functionalization. In recent years, HCPs have shown great application potential in solid phase extraction. Given their high specific surface area, excellent adsorption properties, diverse chemical structures, and easy chemical modification, HCPs have been successfully applied to the extraction of different types of analytes with efficient extraction performance. Based on the chemical structure of HCPs, their target analytes, and the adsorption mechanism, HCPs can be classified as hydrophobic, hydrophilic, and ionic species. Hydrophobic HCPs are usually constructed as extended conjugated structures by overcrosslinking aromatic compounds as monomers. Common monomers include ferrocene, triphenylamine, triphenylphosphine, etc. This type of HCPs shows good adsorption effects on nonpolar analytes such as benzuron herbicides and phthalates through strong *π-π* and hydrophobic interactions. Hydrophilic HCPs are prepared by introducing polar monomers or crosslinking agents, or by modifying polar functional groups. This type of adsorbent is commonly used to extract polar analytes such as nitroimidazole, chlorophenol, tetracycline, etc. In addition to hydrophobic forces, polar interactions, such as hydrogen-bonding and dipole-dipole interactions, also occur between the adsorbent and analyte. Ionic HCPs are mixed-mode solid phase extraction materials formed by introducing ionic functional groups into the polymer. Mixed-mode adsorbents usually have a dual reversed-phase/ion-exchange retention mechanism, which helps control the retention behavior of the adsorbent by adjusting the elution strength of the eluting solvent. In addition, the extraction mode can be switched by controlling the pH of the sample solution and eluting solvent. In this manner, matrix interferences can be removed while the target analytes are enriched. Ionic HCPs present a unique advantage in the extraction of acid-base drugs in water. The combination of new HCP extraction materials with modern analytical techniques, such as chromatography and mass spectrometry, has been widely used in environmental monitoring, food safety, and biochemical analyses. In this review, the characteristics and synthesis methods of HCPs are briefly introduced, and the application progress of different types of HCPs in cartridge-based solid phase extraction is described. Finally, the future outlook of HCP applications is discussed.

固相萃取(SPE)是一种经典的样品前处理方法,具有溶剂用量少、成本低、时间短、富集和回收率高、易于实现自动化等优点,萃取材料是固相萃取技术的关键。传统萃取材料如硅胶和大孔树脂,由于吸附容量较低、选择性较差,应用受到限制^[1]^。随着材料科学与样品前处理技术的交叉发展,许多新型材料,如石墨烯^[2]^、氮化碳^[3]^、金属有机骨架^[4]^、多孔有机聚合物(POPs)^[5,6]^等在样品前处理研究中得到应用。然而,开发低成本、高效、高选择性的固相萃取吸附材料仍是推动分析化学样品前处理技术发展的关键。

POPs是一类由C、H、O、N等轻质元素通过共价键连接而成的多孔材料,具有极高的比表面积和优异的吸附性能。根据结构和晶体性质不同,POPs可分为共价有机框架(COFs)、共轭微孔聚合物(CMPs)、共价三嗪基框架(CTF)、多孔芳香框架(PAFs)、固有微孔聚合物(PIMs)和超交联多孔有机聚合物(HCPs)等^[7]^。然而大多数POPs存在原料和催化剂价格昂贵、制备条件苛刻、产率低等缺点。作为有机多孔材料家族成员之一,HCPs合成方法简便,单体选择性广,溶剂和催化剂较为廉价且反应效率高,在柱固相萃取吸附剂领域引起了广泛关注^[8]^。本文综述了HCPs的合成方法,以及HCPs用作柱固相萃取吸附材料在食品、环境等领域中痕量污染物分析中的应用,并对其发展前景做出了展望。

## 1 HCPs的合成方法

HCPs的合成方法有后交联法、一步缩聚法和外交联法3种^[9]^。

后交联法是将聚合物前体溶解在溶剂中,使聚合物前体的分子链解开并呈舒展状态,然后在路易斯酸的催化作用下通过傅-克反应形成了大量的刚性桥键,将聚合物前体交联起来形成聚合物网络结构(图1a)^[10]^。常用的聚合物前体有聚苯乙烯基或杂环链状聚合物(聚砜、聚芳酯、聚苯胺、聚吡咯等),交联剂通常为双官能化苄基氯代芳烃或醚,如二甲醇缩甲醛(FDA)、1,4-双(氯甲基)苯、4,4'-双(氯甲基)联苯、三氯甲基均三甲苯、对二氯甲基苯基丁烷等,交联剂的长度和刚度可以对产物的结构、比表面积和孔隙率起到调控作用^[11,12]^。

**图1:**
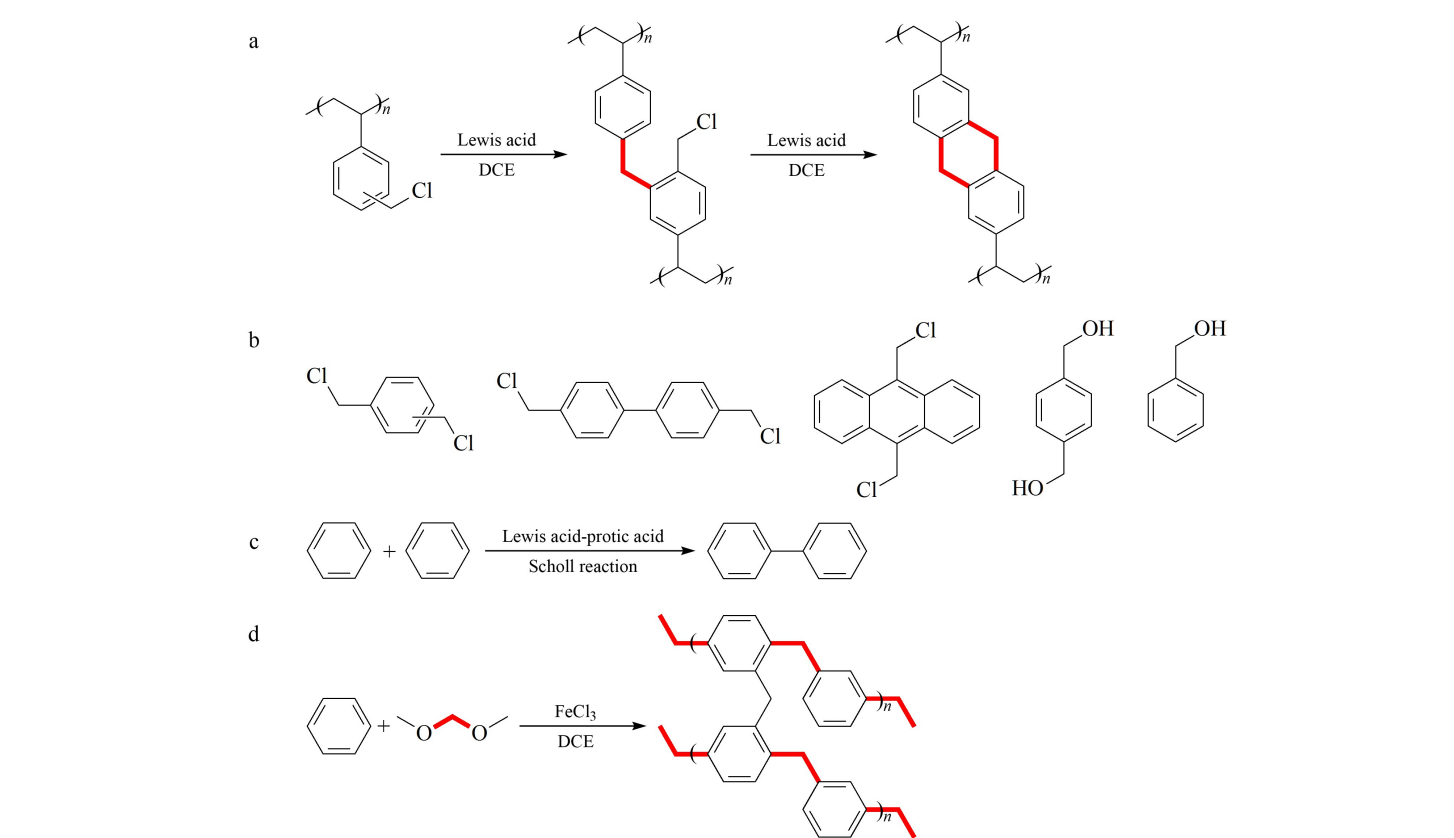
HCPs的制备方法示意图

一步缩聚法是利用功能化小分子作为反应单体,通过一步傅-克反应自缩聚或共缩聚制备HCPs,较为成熟的路线有3种:(1)以双氯甲基芳香化合物包括1,4-双(氯甲基)苯、4,4'-双(氯甲基)联苯和9,10-二氯甲基蒽(图1b)为自缩聚功能单体,在Lewis酸催化作用下,双氯甲基芳香单体上的氯甲基与苯环会发生傅-克反应,在单体之间形成刚性的亚甲基桥,随后数量众多的单体在亚甲基桥连接下形成超交联聚合物网络^[13]^; (2)以苯二甲醇或苯甲醇等苄醇类化合物(图1b)为自缩聚功能单体,与氯甲基苯的反应机理相似,在FeCl_3_的催化作用下,通过在单体之间形成刚性的亚甲基桥来制备HCPs^[14]^; (3)可通过Scholl偶联反应来实现,Tan课题组^[15]^以无取代基团的芳香族化合物为自缩聚功能单体,在无水AlCl_3_的催化下,直接消除相邻苯环上的氢原子,形成了C-C桥键并连接两个苯环,实现了单体的聚合并形成超交联聚合物(图1c)。

外交联法是以多苯环芳香族化合物如苯、联苯、三联苯等为单体,FDA为外交联剂,在单体之间形成刚性的亚甲基桥键,这些桥键将单体深度交联就形成了具有丰富孔道结构和高比表面积的超交联聚合物网络(图1d)^[16⇓-18]^。与其他方法相比,外交联法具有单体来源广泛、原料廉价、反应条件温和、可大规模生产、比表面积和孔径可调等优点。

## 2 HCPs在柱固相萃取中的应用

柱固相萃取是将吸附剂填充于聚丙烯或不锈钢小柱内,再进行活化、上样、净化和洗脱等过程的萃取技术。当样品溶液通过固相萃取柱时,吸附剂通过反相作用(*π-π*作用、疏水作用)、氢键或离子交换等作用力选择性地保留目标化合物,其他组分则透过吸附剂流出小柱,然后用洗脱能力较强的溶剂体系选择性地把目标物洗脱下来,实现对复杂样品的分离、纯化和富集。开发比表面积大、稳定性好、吸附能力强、传质速度快的吸附剂是柱固相萃取领域的研究热点。本综述根据HCPs的骨架结构、目标分析物的性质和萃取机理,将HCPs分为疏水型、亲水型和离子型3类,详细介绍了其在柱固相萃取吸附剂中的应用研究进展。

### 2.1 疏水型HCPs在柱固相萃取中的应用

疏水型HCPs多以芳香化合物为单体,发生超交联后形成的HCPs有延展的共轭结构并通过强*π-π*作用、疏水作用对芳香类目标分析物产生良好的吸附效果。Dmitrienko等^[19]^将超交联聚苯乙烯作为吸附剂用于磺胺类药物的萃取,吸附效率达87%~96%,灵敏度提高了60倍以上,良好的萃取和检测效果归因于其更大的比表面积、较强的*π-π*作用和疏水作用。Wang等^[20]^以二茂铁为单体,通过一步缩聚法制备了两种二茂铁基HCPs,即Fc-DCX-NOP和Fc-BCMBP-NOP。由于二茂铁含有芳香环戊二烯,超交联后可以形成延展的共轭结构,因此对氯酚类化合物(CPs)表现出良好的吸附能力,以Fc-BCMBP-NOP为吸附剂,建立了SPE-高效液相色谱(HPLC)-紫外检测(UV)分析方法测定自来水、红茶饮料和桃汁样品中的CPs,该方法获得了较低的检出限(0.04~0.20 ng/mL)、较宽的线性范围(0.35~80.0 ng/mL)、良好的回收率(87.6%~119%)和重复性(相对标准偏差(RSD)为3.11%~7.83%)。另外,该材料对苯脲类、邻苯二甲酸酯和氨基甲酸酯类化合物也表现出良好的萃取效果。

在芳香单体中引入氮、磷等杂原子,如三苯胺、三苯基膦等单体制备的HCPs不但有延展的共轭结构,还可以与分析物产生氢键作用,Wang课题组^[21⇓⇓-24]^合成了4种HCPs,并对不同基质中的苯脲类农药进行了萃取和检测。他们^[21]^以三苯胺与二氯乙烷的傅-克反应制备了超交联多孔有机聚合物PPTPA, PPTPA中的共轭体系和氮原子可通过*π-π*作用、疏水作用及氢键作用对水、牛奶和番茄汁样品中的苯脲类农药选择性高效萃取,以PPTPA为吸附剂建立的SPE-HPLC-UV分析方法获得了较低的检出限和优于市售吸附剂的回收率。随后该课题组^[22]^以三苯基膦和苯为单体,FDA为外交联剂,制备了超交联多孔有机聚合物Ph-PPh_3_-KAP。Ph-PPh_3_-KAP有扩展的*π*-电子体系、高度稳定的共轭结构和磷原子,通过较强的*π-π*作用、氢键作用对苯脲类农药表现出快速吸附/解吸动力学和大的吸附容量,以Ph-PPh_3_-KAP为吸附剂,结合SPE-HPLC-UV技术对湖水、黄瓜和番茄样品中的苯脲类农药进行萃取和检测,线性范围为0.1~100 ng/mL,检出限为0.01~0.02 ng/mL,加标回收率为80.8%~118%。外交联剂中含有苯环时,苯环作为苯基桥可以引入到HCPs中,得到具有扩展的共轭体系的HCPs,对芳香类污染物有较好的萃取效果。Wang课题组^[23,24]^分别以吡咯、三苯胺为单体,对二甲氧基苯(DMB)为外交联剂,合成得到两种HCPs(Py-DMB-HCP和HCTPA)。它们具有大的比表面积、延展的共轭体系以及能产生氢键作用的氮原子,这种吸附剂可高效萃取苯脲类农药。随后该课题组^[25]^以三苯胺为单体,FDA为交联剂合成了超交联聚合物PTPA,以基于PTPA的SPE-HPLC-质谱(MS)分析方法对桃汁、绿茶饮料和番茄样品中的4种CPs进行了萃取和检测,其精密度和检出限优于大多数其他方法。Zhang等^[26]^以三苯基硅烷、三苯胺和三苯基膦为单体,对苯二甲酰氯为交联剂,通过傅-克酰化反应,合成了3种HCPs(命名为HCP-TPA、HCP-TPS和HCP-TPP),这3种HCPs的结构中含有氢键受体位点和大共轭体系,对CPs物质吸附效果较好,其中吸附效果较强的HCP-TPS被用作吸附剂从水、蜂蜜和白桃饮料中萃取CPs,结果显示该方法具有较宽的线性范围,较低的检出限和优于商品化吸附材料(包括C_18_、石墨化炭黑和碳纳米管)的回收率。

### 2.2 亲水型HCPs在柱固相萃取中的应用

由于单体的芳香性,大多数HCPs具有较强的疏水性,这使得它们在保留极性化合物和在水性基质中的应用受到限制。为了解决这一问题,可通过引入极性单体或交联剂,或以极性官能团后修饰来改善HCPs的亲水性^[27]^。

Xu等^[28]^以天然化合物山奈酚为单体, 4,4'-双(氯甲基)联苯(BCMBP)为交联剂,在FeCl_3_催化下,通过一步缩聚法合成了HCP-Kae1-24h。山奈酚的多羟基结构使其具有亲水亲油平衡特性,对极性硝基咪唑类化合物(NDZs)如甲硝唑、罗硝唑、塞克硝唑、二甲硝咪唑和奥硝唑有较强的吸附效果。将其作为吸附剂建立的SPE-HPLC分析方法用于水、蜂蜜和鱼肉中NDZs药物残留的测定,显示出较低的检出限、较高的准确度和良好的精密度。随后该课题组^[29]^以天然的生物质芹菜素为单体,2,4,6-三(溴甲基)均三甲苯为交联剂,合成了一种亲水型API-HCP,芹菜素结构中的苯环、羰基和羟基使其成为CPs的高效吸附剂。基于API-HCP的柱固相萃取技术,建立了测定水和蜂蜜样品中CPs的分析方法,获得了更低的检出限和接近100%的回收率。采用类似的方案,该课题组^[30]^将1,3,5-三苯基苯(TPB)与极性单体L-酪氨酸(L-Tyr)反应,制备了酪氨酸功能化的新型超高交联聚合物HCP@Tyr。L-Tyr的-NH_2_、-COOH和-OH等多个极性官能团极大地改善了HCP@Tyr的亲水性,对NDZs表现出高吸附容量。基于HCP@Tyr的SPE前处理,利用HPLC-DAD分析方法测定蜂蜜和鸡肉样品中的NDZs时,具有灵敏度高、重复性好的优点。

考虑到硼酸和羟基之间的氢键作用可以增强对含羟基分子的识别和吸附能力,Liu等^[31]^以苯硼酸、1,4-苯二硼酸、4-羟基苯硼酸和4-羧基苯硼酸为极性单体,FDA为交联剂,通过外交联法制备了4种含硼酸基团的HCPs。PBA-HCP对CPs的吸附性能最好,将它作为吸附剂,建立了SPE-HPLC-UV检测方法并成功应用于水和蜜柚饮料中痕量CPs的测定。随后该课题组^[32]^以邻苯二胺、间苯二胺和对苯二胺为极性单体,FDA为外交联剂,通过外交联法制备了3种亲水型HCPs(OPD-HCP、MPD-HCP和PPD-HCP)。苯二氨基HCPs和NDZs都含有芳香结构和丰富的氮原子,可以产生较强的静电相互作用、氢键和亲水作用,在优化条件下对水、果汁、蜂蜜茶和蜂蜜样品中的NDZs进行萃取,该方法线性范围宽(0.07~40.0 ng/mL),回收率高(86.8%~113.3%),检出限低(0.02~0.15 ng/mL),精密度好(RSD小于8.1%)。

除引入极性单体外,引入极性交联剂也可以改善HCPs的亲水性,我们课题组^[33]^以三联苯为单体、三聚氰氯为极性交联剂合成了微孔共价三嗪-三联苯聚合物CTP_CC-TP_(图2)。亲水性三嗪骨架和亲脂性芳香环赋予其亲水-亲脂平衡特性,对四环素类药物(TCs)表现出较好的吸附性能,采用HPLC-UV技术所建立的分析方法检出限为8.0~16.8 μg/kg,线性范围为22.6~1500 μg/kg,回收率为81.3%~98.7%, RSD为3.9%~7.7%。

**图2:**
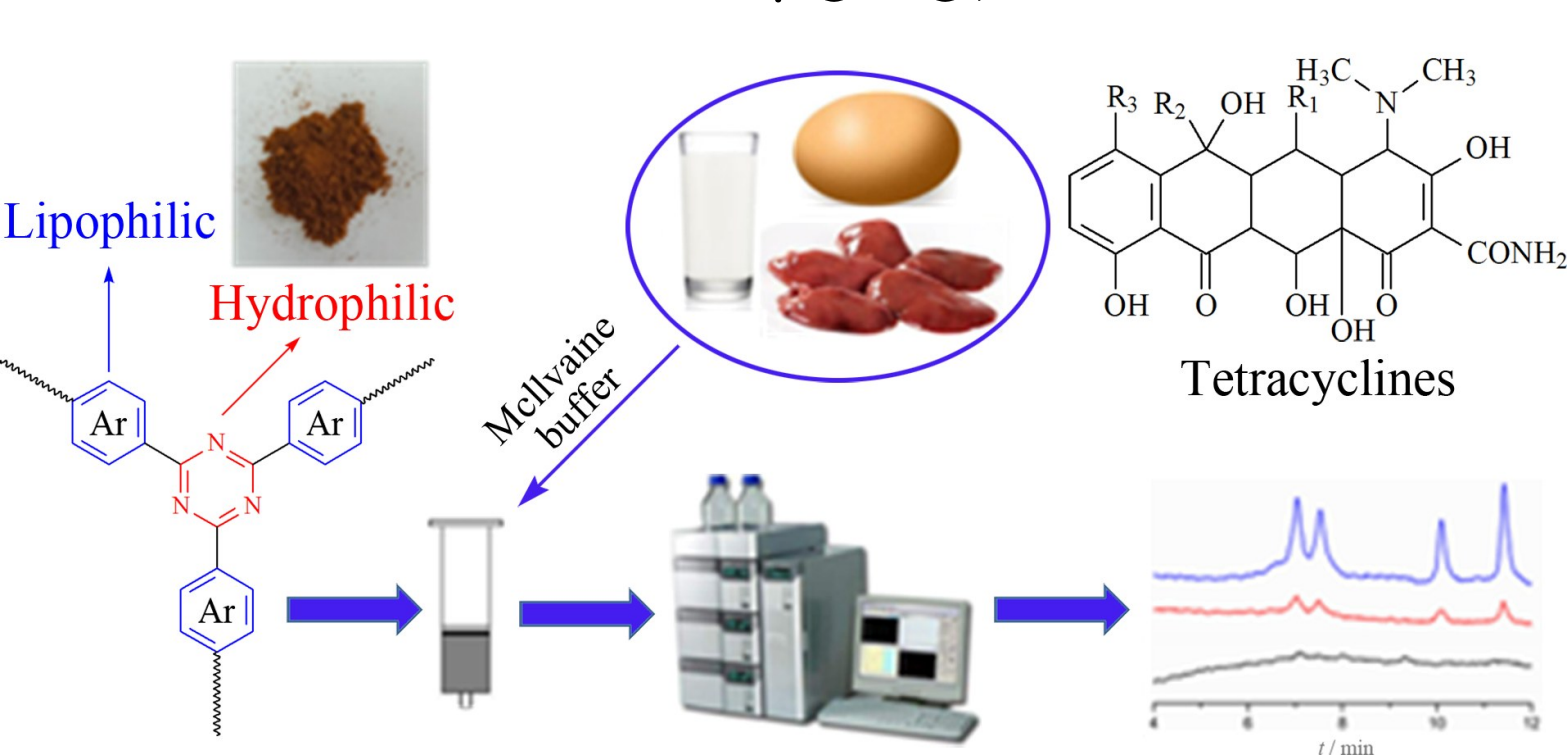
CTP_CC-TP_用于动物源食品中四环素萃取的示意图^[33]^

粒径越小且分布越均匀的HCPs萃取性能越好,Fontanals等^[34]^合成了3种直径为4 μm的亲水性单分散超交联聚合物微球HXLPP,与商品化的吸附剂(LiChrolut EN、HXLGp、HXLGmix、Oasis HLB)有相似的特性(亲水性、比表面积和网络结构),但HXLPP的萃取性能更好,这归因于其更小粒径和单分散的形状特征。该课题组^[35]^还通过后修饰策略制备了一种亲水性超高交联聚合物HXLGp,首先将单体二乙烯基苯(DVB, 2%)和对乙烯基苯苄基氯(VBC, 98%)通过悬浮聚合制得前体树脂,然后再将该前体树脂经后交联合成超交联聚合物HXLGp, VBC在合成过程中会发生水解形成羟基,羟基的存在赋予HXLGp亲水性的同时导致比表面积略有下降,但实验结果表明它对极性化合物依然有较好的萃取性能。

### 2.3 离子型HCPs在柱固相萃取中的应用

离子型HCPs是在聚合物中引入离子官能团,得到混合模式的固相萃取材料,混合模式吸附剂通常具有疏水和离子交换基团,可以通过调节洗脱溶剂的洗脱强度来控制吸附剂对目标物的保留行为,还可以通过控制上样溶液和洗脱溶剂的pH来实现萃取模式的切换,在富集目标分析物的同时去除基质干扰;另一方面,当样品中含有不同种类的目标分析物时,通过优化上样和洗脱条件,可实现多类别目标物的同时萃取和分类洗脱,因此混合模式固相萃取被广泛应用于环境、生物、食品和植物样品中各种酸性或碱性化合物的选择性萃取。

Fontanals等^[36]^将合成的超高交联聚合物树脂(HXLPPs)用乙二胺和哌嗪(piperazine)部分改性,制备了两种具有弱阴离子交换特性的超交联聚合物树脂HXLPP-WAX-EDA和HXLPP-WAX-piperazine。它们具有超高的比表面积(>1000 m^2^/g)、高微孔体积和较小的粒径。洗脱时,洗涤步骤中的甲醇只能除去通过反相作用保留在吸附剂上的碱性和中性化合物,而酸性化合物在静电作用下仍选择性保留。以HXLPP-WAX-EDA和HXLPP-WAX-piperazine为吸附剂,结合离线SPE-LC方法应用于河水中水杨酸、萘啶酸等酸性化合物的选择性萃取,结果显示所测定的酸性化合物回收率达到100%,高于其他吸附材料(HXLPP、Oasis-WAX、Oasis-HLB、Strata-X-AW、Strata-X)。随后该课题组^[37]^以HXLPP-EDA为吸附剂,结合online-SPE-HPLC方法对大量超纯水、河水和污水中的酸性分析物进行选择性萃取和检测,基于高比表面积和小颗粒尺寸的形态特征,HXLPP-EDA材料的吸附容量增加,在线固相萃取模式与离线模式相比效率更高,降低了分析方法的检出限,并获得了良好的线性范围和重现性。

Hu等^[38]^采用沉淀聚合和氨基改性的方法合成了具有高比表面积和离子交换特性的弱阴离子交换超高交联树脂(HXLPP-WAX)(图3a),并结合SPE-HPLC-UV分析方法测定人尿中酸性化合物高香草酸(HVA);结果表明,该方法在2.26~289 mg/L范围内具有良好的线性关系,检出限为0.45 mg/L,平均回收率大于90%,RSD小于4.2%,并成功用于8个健康人尿液样品中HVA的含量测定。Bratkowska等^[39]^通过单体(75% VBC和25% DVB)优化的沉淀聚合法合成球形前体(PP)颗粒,然后将PP通过后交联和季铵化反应合成了两种高比表面积的HCPs(HXLPP-SAXa和HXLPP-SAX_b_),这种HCPs既有微孔,又有可选择性吸附的强阴离子交换功能基团,对水杨酸、氯贝酸、双氯芬酸、布洛芬等酸性化合物有优异的选择性吸附效果。Liang等^[40]^通过一步超交联和季铵化反应合成了强阴离子交换超高交联树脂(HXLPP-SAX)(图3b),该材料具有粒径分布窄、高比表面积的单分散微球特性,与市售的强阴离子交换吸附剂(Oasis MAX)相比,该吸附剂对两性化合物(以依诺沙星和恩诺沙星为代表)的提取具有较高的选择性;该方法线性关系良好(*R*^2^≥0.997,线性范围为10~2000 ng/g),检出限低(2.8~5.1 ng/g),加标回收率高(85.8%~117.9%, RSD≤7.1%),精密度高(日内RSD≤7.7%,日间RSD≤9.4%)。

**图3:**
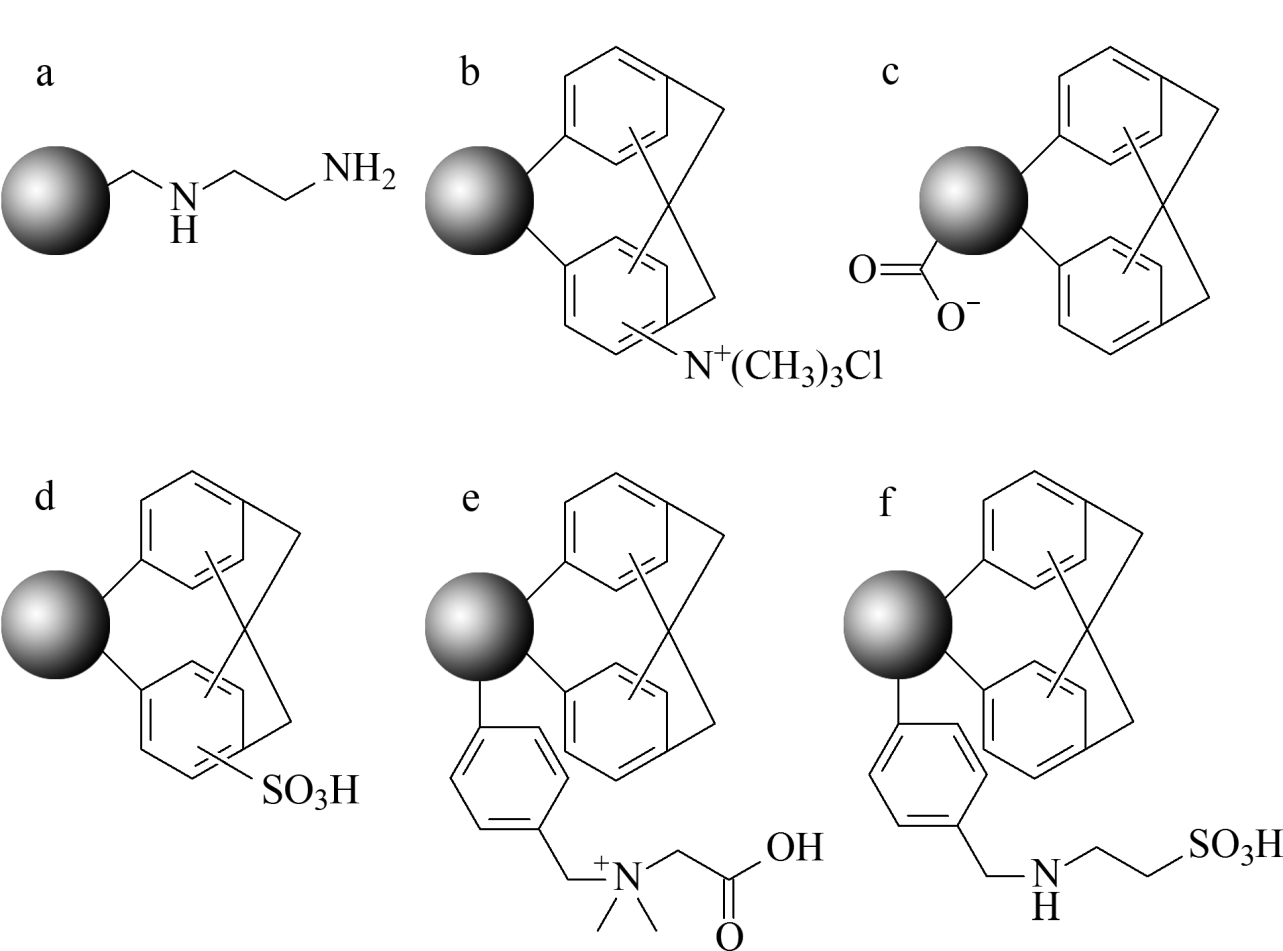
离子型HCPs的结构

Bratkowska等^[41]^合成了弱阳离子超高交联吸附剂HXLPP-WCX,他们首先将单体(10%甲基丙烯酸(MAA)、50% VBC和40% DVB)通过沉淀聚合合成前体聚合物PP,再将PP后交联合成弱阳离子超高交联吸附剂HXLPP-WCX(图3c),弱阳离子特性源于共聚单体甲基丙烯酸的羧酸官能团,可以选择性萃取碱性化合物,该吸附剂成功用于废水中μg/L水平的碱性药物的选择性萃取和定量检测。Xu等^[42]^则将HXLPP磺化制得强阳离子交换型HXLPP-SCX树脂(图3d),所得树脂具有高比表面积和单分散的粒径特征,吸附容量较高,阳离子特性使其对碱性化合物有选择性萃取功能,以HXLPP-SCX为吸附剂结合HPLC用于选择性萃取人血清中的3种碱性嘌呤代谢物,结果显示大多数基质干扰(包括酸性和中性化合物)被消除,而碱性嘌呤化合物被高效选择性萃取。

为实现在同一个固相萃取柱中同时选择性萃取酸性和碱性分析物,Nadal等^[43]^在聚合物中同时引入了阳离子和阴离子交换基团并制得了具有两性离子交换特性的HCPs。该HCPs以聚(二乙烯基苯-乙烯基苄基氯)为前体聚合物,后交联后具有高微孔含量和高比表面积,增加了吸附容量,然后分别以季铵化肌氨酸和牛磺酸修饰得到HXLPP-SAX/WCX(图3e)和HXLPP-WAX/SCX(图3f), HXLPP-SAX/WCX具有更高的回收率和吸附容量,因此被用作固相萃取吸附剂用于河水和污水中酸性和碱性化合物的选择性萃取,结果显示该方法具有优异的选择性和较低的基质效应,大多数化合物的回收率为52%~105%。此外,该方法还显示出优异的灵敏度,用于样品分析时,ng/L级水平的氯贝酸、非诺洛芬、萘普生、美沙酮、美托洛尔等物质均被检测出来,实现了一种吸附剂可同时选择性萃取酸性和碱性化合物。

综上,表1详细总结了用于柱固相萃取的HCPs的制备方法、目标分析物、分析基质和作用机理。

**表1:** 不同HCPs在柱固相萃取中的应用

Sorbent	Monomer	Synthesized method	Analytes	Mechanism	Sample	Ref.
HCPs	styrene	post-crosslinking	sulfanilamides	*π-π*, hydrophobic	river water	[19]
Fc-NOP	ferrocene	one-step polycon- densation	phenylurea herbicides	*π-π*, hydrophobic	tap water, black tea drinks, peach juice	[20]
PPTPA	triphenylamine	one-step polycon- densation	phenylurea herbicides	*π-π*, hydrophobic, hydrogen bonding	water, milk, tomato juice	[21]
Ph-PPh_3_-KAP	PPh_3_, benzene	external crosslinking	phenylurea pesticides	*π-π*, hydrogen bonding	lake water, tomato, cucumber	[22]
Py-DMB-HCP	pyrrole	external crosslinking	phenylurea pesticides	*π-π*, hydrophobic, hydrogen bonding	soybean milk, tomato	[23]
HCTPA	triphenylamine	external crosslinking	phenylurea pesticides	*π-π*, hydrophobic, hydrogen bonding	watermelon, tomato, cucumber	[24]
PTPA	triphenylamine	external crosslinking	chlorophenols	*π-π*, hydrophobic, hydrogen bonding	peach juice, green tea beverage, tomato	[25]
HCP-TPS	triphenylsilane	one-step polycon- densation	chlorophenols	*π-π*, hydrophobic, hydrogen bonding	water, honey, white peach beverage	[26]
HCP-Kae1-24h	kaempferol	one-step polycon- densation	nitroimidazoles	weak *π-π*, hydropho- bic, hydrogen bonding	water, honey, fish meat	[28]
API-HCP	apigenin	one-step polycon- densation	chlorophenols	hydrogen bonding, halogen bonding, *π-π* stacking	water, honey	[29]
HCP@Tyr	TPB, L-Tyr	external crosslinking	nitroimidazoles	*π-π*, hydrogen bond- ing, hydrophilic	honey, chicken muscle	[30]
PBA-HCP	PBA	external crosslinking	chlorophenols	hydrophilic, hydrogen bonding	water, honey-pomelo beverage	[31]
MPD-HCP	MPD	external crosslinking	nitroimidazoles	electrostatic, hydrogen bonding, hydrophilic	water, peach juice, honey tea, honey	[32]
CTP_CC-TP_	terphenyl, cyanuric chloride	one-step polycon- densation	tetracycline	hydrophilic, hydropho- bic, hydrogen bonding	bovine milk, egg, chicken liver	[33]
HXLPP	VBC, DVB	post-crosslinking	polar pollutants	hydrophilic, hydrogen bonding, polar	ultrapure, mineral, tap and Ebre river water	[34]
HXLGp	2% DVB, 98% VBC	post-crosslinking	polar pollutants	hydrophilic, hydrogen bonding, polar	tap, river water	[35]
HXLPP-WAX-EDA HXLPP-WAX- piperazine	75%VBC, 25%DVB	post-crosslinking	acidic com- pounds	reversed-phase, weak anion exchange	Ebre river water, efflu- ent waste water	[36]
HXLPP-WAX-EDA	75% VBC, 25% DVB	post-crosslinking	acidic com- pounds	reversed-phase, weak anion exchange	ultrapure, river, effluent sewage water	[37]
HXLPP-WAX	VBC, DVB	post-crosslinking	homovanilic acid	reversed-phase, weak anion exchange	human urine	[38]
HXLPP-SAX_a_, HXLPP-SAX_b_	75% VBC, 25% DVB	post-crosslinking	acidic pharma- ceuticals	reversed-phase, strong anion exchange	Ebre river water, sew- age water	[39]
HXLPP-SAX-N(CH_3_)	VBC, DVB	post-crosslinking	fluoroquinolone drugs	reversed-phase, strong anion exchange	milk	[40]
HXLPP-WCX-MAA	10% MAA, 50% VBC and 40% DVB	post-crosslinking	basic pharma- ceuticals	reversed-phase, weak cation exchange	Ebre river water, waste water	[41]
HXLPP-SCX	VBC, DVB	post-crosslinking	hypoxanthine, xanthine and inosine	reversed-phase, strong cation exchange	serum	[42]
HXLPP-SAX/WCX, HXLPP-WAX/SCX	poly(divinyl- benzene-co-vinyl- benzylchloride)	post-crosslinking	acidic and basic drugs	reversed-phase, ion exchange	river water, effluent waste water	[43]

PPh_3_: triphenylphosphine; TPB: triphenylbenzene; L-Tyr: L-tyrosine; PBA: phenylboric acid; MPD: *m*-phenylenediamine; VBC: *p*-vinylbenzyl chloride; DVB: divinylbenzene; MAA: methacrylic acid.

## 3 结论和展望

作为一种新型吸附剂,HCPs广泛应用于各种分析物的萃取和检测。疏水型HCPs主要用于苯脲类、苯甲酰脲类、邻苯二甲酸酯类、氯酚等芳香污染物的萃取和检测;亲水型HCPs主要用于NDZs、TCs等极性化合物的萃取和检测;离子型HCPs主要用于酸碱化合物的选择性萃取和检测。随着社会经济的发展,更多类型的痕量污染物层出不穷,具有特异识别作用的功能化HCPs的开发显得尤为必要;此外,柱固相萃取分离过程繁琐且耗时长,需要特定的检测设备和环境,不能适应多样的检测环境。因此未来应开发具有特异识别机理的功能化HCPs,并与现代样品前处理技术(如磁性固相萃取、固相微萃取)结合对复杂体系中更多类型的目标分析物进行快速萃取和检测,更多类型的HCPs和分析方法在未来将会不断出现。
